# Investigating the Black Birth Experience: A Race-Stratified Analysis of Preterm Birth Risk and Exposure to Metropolitan Statistical Area-Level Police-Related Deaths, US 2018–2019

**DOI:** 10.1007/s11524-024-00871-x

**Published:** 2024-05-16

**Authors:** Lauren Dyer, Jé Judson, Jaquelyn L. Jahn, Maeve Wallace

**Affiliations:** 1 Mary Amelia Center for Women’s Health Equity Research, Department of Social, Behavioral, and Population Sciences, Tulane University School of Public Health and Tropical Medicine, 1440 Canal St., New Orleans, LA 70112 USA; 2 Department of Epidemiology and Biostatistics, Drexel University Dornsife School of Public Health, Nesbitt Hall, 3215 Market St., Philadelphia, PA USA; 3 Center for Antiracism Research for Health Equity, School of Public Health, University of Minnesota, 2001 Plymouth Ave Ste. 106, Minneapolis, MN 55411 USA

**Keywords:** Preterm birth, Police violence, Health equity

## Abstract

Police-related violence may be a source of chronic stress underlying entrenched racial inequities in reproductive health in the USA. Using publicly available data on police-related fatalities, we estimated total and victim race-specific rates of police-related fatalities (deaths per 100,000 population) in 2018–2019 for Metropolitan Statistical Areas (MSA) and counties within MSAs in the USA. Rates were linked to data on live births by maternal MSA and county of residence. We fit adjusted log-Poisson models with generalized estimating equations and cluster-robust standard errors to estimate the relative risk of preterm birth associated with the middle and highest tertiles of police-related fatalities compared to the lowest tertile. We included a test for heterogeneity by maternal race/ethnicity and additionally fit race/ethnicity-stratified models for associations with victim race/ethnicity-specific police-related fatality rates. Fully adjusted models indicated significant adverse associations between police-related fatality rates and relative risk of preterm birth for the total population, non-Hispanic Black, and non-Hispanic White groups separately. Results confirm the role of fatal police violence as a social determinant of population health outcomes and inequities, including preterm birth.

**Supplementary Information:**

The online version contains supplementary material available at 10.1007/s11524-024-00871-x.

## Background

A long, complex history surrounds the tumultuous relationship between US police agencies and the communities that they serve. From an historical perspective, policing structures have been molded into a sociopolitical tool to enforce social control, at the expense of entrenching inequities [1]. Currently, apprehension toward the police is reaching a fever-pitch due to the various high-profile killings of unarmed civilians with an estimated 15 million to 26 million people in the USA participating in demonstrations over the death of George Floyd in 2020 [2]. The racial component of police-related deaths in the USA merits additional scrutiny because of the undue burden of deaths borne by Black and Hispanic victims. A recent study estimated that Black individuals across all US Metropolitan Statistical Areas (MSAs) were 3.23 times more likely to be killed by the police when compared to White individuals [3]. Another study showed that the Black versus White excess risk of experiencing a police-related death was 3.1, with variation within and across US cities over time [4].

In addition to police-related fatalities, morbidity associated with police encounters has also been noted in public health literature. For instance, one study estimated that individuals who experienced negative encounters with the police had significantly greater odds of having unmet need for mental healthcare when compared to those who reported having no negative encounters with the police [5]. It was also estimated that individuals who were victimized by the police, and those who anticipated future victimization, were more psychologically distressed and depressed when compared to those who had more neutral or positive interactions with the police with disparities persisting along racial lines [6]. Adverse psychological effects—such as depression and self-reporting of poor mental health—have been detected among Black adults who lived in an area where a police officer killed an unarmed Black citizen [7]. In an analysis of data from the New York City Community Health Survey, an increase in the stop rate per 100 non-institutionalized population increased the odds of poor or fair perceptions of one’s general health by 14.8%, and the odds of having an asthma episode within the past year by 17% [8].

The nature of police-related violence calls for the use of an intersectional lens due to the various social domains it encompasses, including not only race, but also gender. The unique social position that Black women inhabit exposes them to gendered racism wherein they experience inequities and discrimination through both their race and their gender [9]. However, the mechanisms behind the inequities experienced by Black women are found not in individual social-behavioral habits, but in underlying racist and misogynistic structural and systemic phenomena which uphold Whiteness and White supremacy [10]. While some of the potential health consequences of police violence stem from personal exposure, Black women who witness or learn about police violence through media or social networks may also experience what Alang et al. term “vicarious marginalization,” described as the marginalizing effect of police maltreatment that is targeted toward others [11]. Distress resulting from either direct or proxy police violence has particular importance with regard to reproductive health since preterm term birth is known to be strongly associated with maternal stress [12]. For instance, Black women have been shown to have an increased risk of both late preterm birth and pregnancy loss following proximate exposure to fatal police violence [13, 14]. A qualitative study of Black pregnant women in New Haven, Connecticut, also found that many reported feeling anxiety and stress when considering future interactions with the police, not only for themselves, but for their future children [15].

Despite growing research on the intersectional consequences of fatal police violence for the health of Black women, to our knowledge, no previous study has examined exposure to police-related fatalities in relation to racial inequities in preterm birth at the level of the Metropolitan Statistical Area (MSA). Not only do MSAs encompass the areas across counties in which people live, work, and travel, but they are also particularly relevant to research on police violence since 85% of police-related deaths occurred within an MSA [3]. Therefore, the aim of this study was to use a comprehensive, rigorously tested, crowd-sourced dataset on police-related deaths to assess the relationship between police-related violence and preterm birth in the total population and within maternal racial/ethnic identity. We hypothesized a significant, positive association between preterm birth and police-related deaths such that persons living in places with higher rates of police-related fatality would be more likely to experience preterm birth.

## Methods

### Study Data and Outcome

This is a retrospective analysis of data from the 2018 and 2019 restricted use natality files provided by the National Center for Health Statistics. These data include live birth records for every birth in the USA, with a geographic identifier for maternal county of residence. We used the Census Bureau’s Core-Based Statistical Area to Federal Information Processing System (FIPS) County Crosswalk to identify births occurring in Metropolitan Statistical Areas [16]. A Metropolitan Statistical Area (MSA) contains at least one urbanized core area of 50,000 or more inhabitants and adjacent communities with a high degree of economic and social integration with the core. MSAs are groups of counties or county equivalents, enabling full spatial linkage between birth record identifiers for maternal county of residence and the MSA that it falls within. There are 384 MSAs in the USA. We restricted this analysis to births in MSAs where there was at least 1 police-involved fatality in the study period (*n* = 324; see Supplemental Table 1 for a complete listing) and used the data on gestational age at birth from the birth records to identify all cases of preterm birth (those occurring at < 37 weeks). Our final sample size was 5,944,472 births after excluding records where outcome or covariate data was missing.

### Fatal Police Violence

Data on fatal police violence came from Fatal Encounters, an open-source database of systematically identified cases of police-involved fatalities gleaned from online media reports and public records [17]. This dataset is verified by paid researchers and overcomes limitations of relying on administrative sources—such as vital statistics—which have been found to substantially underestimate the number of police-involved fatalities and lack accurate contextual details, such as arrest type and location [18–20]. Fatal Encounters has been endorsed by the Bureau of Justice Statistics for identification of police-involved fatalities due to the accuracy of their methods [21].

In following with the Fatal Encounters inclusion criteria utilized by Edwards et al. [22], we coded police-related fatalities as those where the victim was asphyxiated/restrained, beaten/bludgeoned with an instrument, confronted with a chemical agent/pepper spray, shot by a firearm, experienced a medical emergency, or tasered. We excluded deaths classified as suicide and deaths due to burning/smoke inhalation, drowning, drug overdose, falling from a height, other, stabbing, undetermined. In addition to these criteria, we also included cases where the mechanism of death involved a vehicle. Vehicle deaths are of particular concern in the consideration of police-related violence since traffic stops are one of the most common forms of police interaction. There are glaring inequities in the outcomes of traffic stops as Black and Hispanic individuals have been estimated to be more likely to be pulled over by the police and to experience the threat or use of physical force by the police during traffic stops when compared to non-Hispanic White individuals [23]. As such, police-related deaths involving a vehicle may be an attempt for civilians to avoid these traffic interactions altogether, which then leads to car chases and the potential for additional morbidity and mortality in the event of a crash. Between 2000 and 2019, 60% of Black women and girls in the Fatal Encounters dataset were killed in a police-related encounter that led to a car crash [24]. The majority of these incidents were collateral damage, whereby the decedent was not the intended target of the police activity but was rather a passenger in the car or a bystander in another vehicle or on foot [24]. Police-related deaths of this type thus may have psychologically traumatic effects—particularly among the Black population—similarly to other causes of police-related death for individuals who reside nearby. For this reason, we included vehicular deaths in our final case definition, in addition to the other criteria previously listed.

Fatal Encounters provides the latitude and longitude of each incident. These data were used to map all police-related deaths in 2018–2019 and were spatially linked to the 2018 MSA shapefile (US Census Bureau) in ArcMap 10.7. Counts of police-related fatalities were aggregated to the MSA, and year-specific population denominators (data obtained from the US Census Bureau’s American Community Survey 5-Year Estimates) were used to estimate annual MSA-level rates of police-related fatalities per 100,000 residents. We repeated the aggregation for race group-specific counts and rates using the victim race/ethnicity variable in Fatal Encounters to identify non-Hispanic White and non-Hispanic Black decedents.

Given the statistical infrequency of police-related fatalities and the instability of fatality rates estimated in MSAs with smaller populations (especially when racially stratified), we calculated empirical Bayes smoothed fatality rates (per 100,000 population total, and by race) by applying the smoothing tool in GeoDa version 1.12.1.161. The empirical Bayes smoothing approach computes a weighted average between the raw estimate for each MSA and the sample average, with weights proportional to the underlying population at risk [25, 26]. As a result, spurious outliers (MSAs with small populations and large rate variance instability) are adjusted to a greater degree than larger MSAs with more stable rates.

After linkage of annual police-related fatality rates to birth records by MSA and county FIPS codes and year, we categorized exposures into tertiles of low, medium, and high levels of police-related fatality based on the whole sample distribution to further reduce bias from extreme outliers and to avoid assumptions of a linear trend.

### Covariates

We identified a set of covariates a priori for their relationship to fatal violence and risk of preterm birth. Individual-level covariates obtained from birth records included maternal age (< 20, 20–24, 25–29, 30–34, ≥ 35), parity (nulliparous, multiparous), whether or not the birth was a singleton, and health insurance type as a marker of socioeconomic position (Medicaid, private insurance, other). Area-level characteristics included economic indicators from the American Community Survey’s (ACS) 2018 and 2019 5-year estimates: the Gini index of income inequality and child poverty rate. We also included the annual rate of violent crime (incidents per 1000 population) in each MSA or county, using aggregated counts obtained from the Federal Bureau of Investigation’s Uniform Crime Reporting System and population denominators from the ACS.

### Primary Statistical Analysis

We first described maternal sociodemographic and MSA characteristics overall and by preterm birth status in addition to the distribution of police-related fatality rates across all MSAs with at least 1 incident from 2018 to 2019. We fit log-Poisson models with generalized estimating equations and MSA cluster-robust standard errors to estimate the relative risk of preterm birth associated with the middle and highest tertiles of police-related fatality compared to the lowest tertile. Models included state and year fixed effects, in addition to all of the individual and area-level covariates detailed above. We included a test for racial and ethnic heterogeneity in the association between police-related fatality rates and preterm birth by including a multiplicative interaction term in the fully adjusted model stratified by maternal race/ethnicity. Finally, we explored associations using race/ethnicity-specific rates of police-related violence victimization to estimate risk of preterm birth in race/ethnicity-stratified models. These analyses were conducted in SAS 9.4.

### Sensitivity Analyses

While previous research has shown that most police-related fatalities occur in metropolitan areas [3], there may be significant sociodemographic, economic, and other contextual differences *within* MSAs that are relevant to the association between police violence and preterm birth. In particular, previous studies have shown that city- and neighborhood-level risk of fatal police violence among Black populations is greater in areas that experience higher racial residential segregation [27, 28]. This may be due to the Racial Threat Hypothesis, which posits that police are more punitive in urban areas with higher concentrations of Black residents [29]. In order to explore potential heterogeneity within MSAs, we additionally estimated county-level police-related fatality rates among Black victims for every county in the USA that is within an MSA. We applied the rate smoothing procedure described above, and fit a fully adjusted model to estimate the relative risk of preterm birth among Black persons associated with tertiles of the county-level Black police-related fatality rates.

## Results

**Fig. 1:**
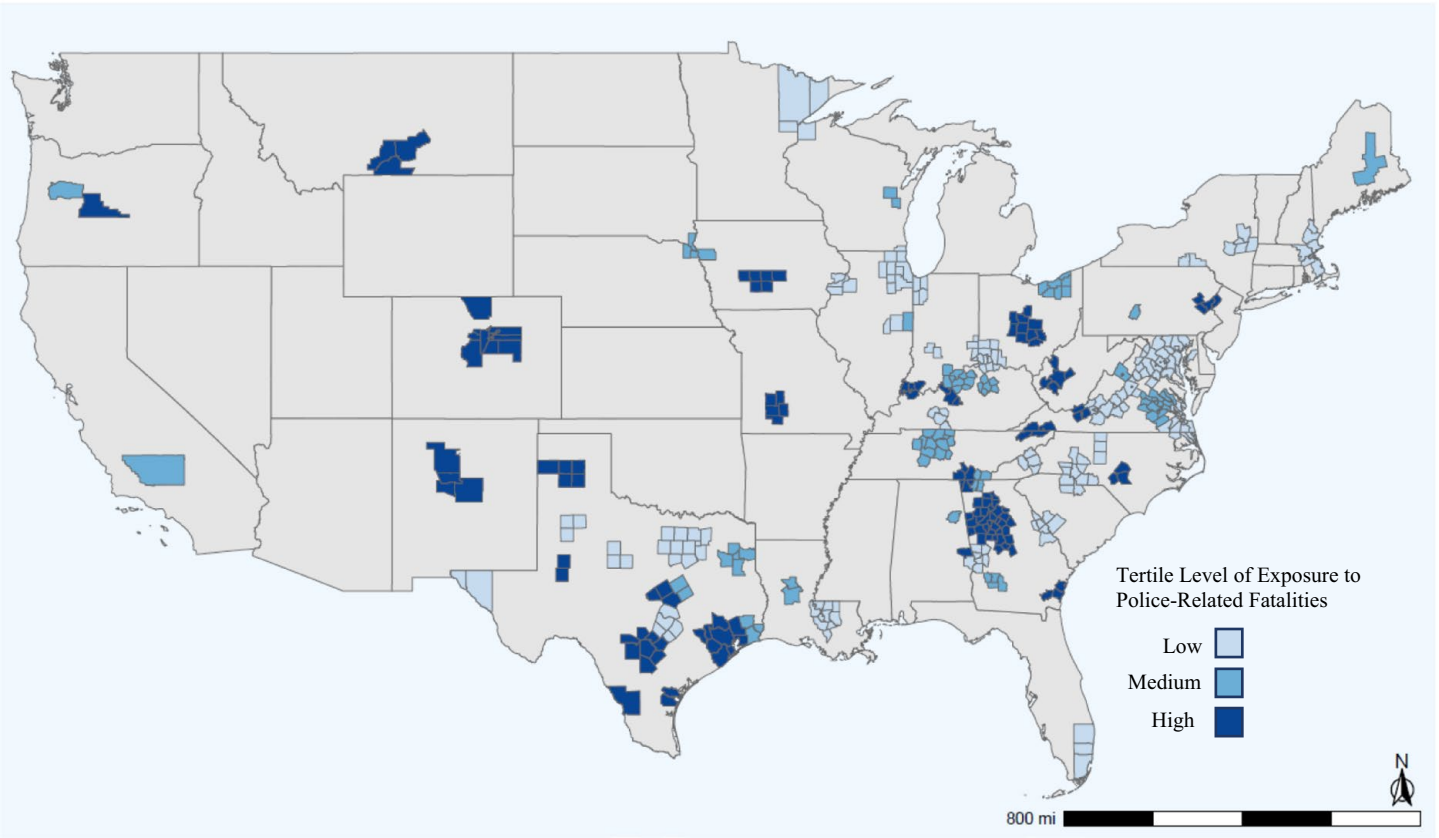
Map of county-level tertiles of fatal police violence in US Metropolitan Statistical Areas (MSA) with at least one police-related fatality from 2018 to 2019

During 2018–2019, there were 5,944,472 births that occurred in MSAs with at least 1 fatal incident of police-related violence. Approximately 10% of births were preterm (*n* = 601,317). The distribution of these births by maternal sociodemographic characteristics is shown in Table 1. On average across MSAs, there were 0.56 police-related fatalities per 100,000 residents, ranging from 0.19 to 1.40 (Table 2). Black pregnant people were exposed to an average police violence rate of 0.55 deaths per 100,000 MSA residents, and White pregnant people were exposed to an average police violence rate of 0.57 deaths per 100,000 MSA residents (Table 1). Fatal police violence rates in which the victim was Black averaged 1.03 deaths per 100,000 Black residents, compared to a White victimization rate of 0.35 deaths per 100,000 White residents. The MSA-level tertiles of racially stratified police-related deaths are depicted in Fig. 1.

**Table 1:** Characteristics of births and MSA of maternal residence for the total population and by preterm birth status, all US MSAs with at least one incident of police-related death, 2018–2019

	All births (*n* = 5,944,472)	Preterm births (*n* = 601,317)	Full-term births (*n* = 5,367,406)
	*N* (%)	*N* (%)	*N* (%)
Race/ethnicity			
Hispanic	1,540,058 (25.91)	151,083 (9.81)	1,384,459 (90.19)
Non-Hispanic Black	952,544 (16.02)	134,720 (14.14)	813,128 (85.86)
Non-Hispanic White	2,823,248 (47.49)	253,799 (8.99)	2,546,233 (91.01)
Non-Hispanic other	628,622 (10.57)	58,136 (9.25)	568,717 (90.75)
Maternal age			
< 20	258,502 (4.30)	26,832 (10.38)	230,099 (89.62)
20–24	1,051,401 (17.50)	101,918 (9.69)	942,468 (90.30)
25–29	1,687,184 (28.09)	158,731 (9.41)	1,518,384 (90.59)
30–34	1,809,916 (30.13)	17,444 (9.64)	1,625,764 (90.36)
≥ 35	1,199,537 (19.97)	142,930 (11.92)	1,050,691 (88.08)
Singleton pregnancy			
Yes	5,804,605 (96.64)	481,127 (8.29)	5,289,769 (91.71)
No	201,935 (3.36)	123,738 (61.28)	77,637 (38.70)
Nulliparous			
Yes	2,318,976 (38.61)	219,833 (9.48)	2,086,768 (90.52)
No	3,687,564 (61.39)	385,032 (10.44)	3,280,638 (89.55)
Insurance type			
Medicaid	2,452,868 (40.84)	276,667 (11.28)	2,176,201 (88.72)
Private insurance	3,061,982 (50.98)	280,910 (9.17)	2,781,072 (90.83)
Other insurance type	491,690 (8.19)	47,288 (9.62)	444,402 (90.38)
MSA of maternal residence	Mean (standard deviation, median, interquartile range)	Mean (standard deviation, median, interquartile range)	Mean (standard deviation, median, interquartile range)
Total police-involved fatality rate (deaths per 100,000 residents)	0.56 (0.24, 0.31, 0.55)	0.57(0.24, 0.30, 0.55)	0.56 (0.24, 0.31, 0.55)
Child poverty rate (%)	18.24 (4.96, 18.30, 5.0)	18.55 (5.06, 18.60, 4.60)	18.20 (4.94, 18.20, 5.10)
Gini index of income inequality	47.06 (2.32, 46.85, 2.90)	47.07 (2.27, 46.92, 2.72)	47.06 (2.32, 46.82, 2.91)
Violent crime rate (per 1000 residents)	3.95 (1.49, 3.70, 1.76)	4.00 (1.54, 3.75, 1.79)	3.94 (1.49, 3.70, 1.76)

Row percents are provided for each demographic variable to demonstrate the prevalence of preterm birth status among each category

**Table 2:** Distribution of smoothed police-related fatality rates (deaths per 100,000 residents) overall and by victim race/ethnicity, all US MSAs with at least 1 incident, 2018–2019

	All births			NH Black births			NH White births		
	Mean (SD)	Median	Min–max	Mean (SD)	Median	Min–max	Mean (SD)	Median	Min–max
Total police-involved fatality rate	0.56 (0.24)	0.55	0.19–1.40	0.55 (24)	0.53	0.22–1.40	0.57 (0.25)	0.55	0.22–1.40
Police-involved fatality rate where victim was non-Hispanic Black	1.14 (0.69)	0.93	0.16–5.29	1.04 (.59)	0.85	0.16–5.29	1.17 (0.75)	0.93	0.16–5.29
Police-involved fatality rate where victim was non-Hispanic White	0.33 (0.19)	0.31	0.03–1.39	0.32 (0.19)	0.29	0.03–1.39	0.35 (0.19)	0.31	0.03–1.39

There were statistically significant adverse associations identified for the total population, non-Hispanic Black, and non-Hispanic White groups separately. Fully adjusted models indicated a significant 3% relative risk of preterm birth among all births given exposure to the medium tertile of police-related fatality rate for victims of any race (Table 3). When analyzed by maternal race, the relative risk of preterm birth given this same exposure was 4% among White births and 6% among Black births with results being significant at the 95% confidence level. Results from the models stratified by victim race indicated that residing in MSAs with high levels of police-related fatalities involving non-Hispanic White victims was associated with elevated risk for preterm birth among both non-Hispanic Black and non-Hispanic White pregnant people, but there was no association between the non-Hispanic Black victimization rate and preterm birth for either racial/ethnic group (Table 4).

**Table 3:** Adjusted* relative risk of preterm birth and 95% confidence intervals associated with tertiles of police-related fatality rate overall and by maternal race/ethnicity, all MSAs with at least 1 incident, 2018–2019

	All births		NH Black		NH White	
	RR	95% CI	RR	95% CI	RR	95% CI
Tertile						
Low	Ref		Ref		Ref	
Medium	1.03	1.01–1.06	1.06	1.02–1.09	1.04	1.01–1.07
High	1.03	0.99–1.05	1.02	.98–1.06	1.03	1.01–1.06

*Adjusted model covariates included state, year, child poverty rate, Gini index of income inequality, violent crime rate per 1000 residents, maternal age, insurance type, nulliparity, and singleton status

**Table 4:** Adjusted* relative risk and 95% confidence intervals for tertiles of racially stratified fatal police violence rates and by maternal race/ethnicity, all MSAs with at least 1 incident, 2018–2019

	NH Black		NH White	
	RR	95% CI	RR	95% CI
Tertile of police-related fatality rate where victim was non-Hispanic Black				
Low	Ref			
Medium	.95	.86–1.04	.98	.95–1.00
High	1.01	.98–1.05	.99	.97–1.01
Tertile of police-related fatality rate where victim was non-Hispanic White				
Low	Ref		Ref	
Medium	1.05	1.02–1.08	1.06	1.04–1.09
High	1.01	.97–1.05	1.05	1.02–1.08

*Adjusted model covariates included state, year, child poverty rate, Gini index of income inequality, violent crime rate per 1000 residents, maternal age, insurance type, nulliparity, and singleton status

## Discussion

The results of this analysis suggest a potentially measurable effect of racialized police violence on reproductive health outcomes in surrounding communities. The rate of fatal police violence against non-Hispanic Black persons was more than threefold higher, on average, than the rate of fatal police violence against non-Hispanic White persons, and preterm birth occurred more than 1.5 times more frequently among non-Hispanic Black pregnant people compared to their non-Hispanic White counterparts. Modeled estimates, however, suggested that the strongest associations between fatal police violence and preterm birth risk were in MSAs with high levels of non-Hispanic White victimization and among non-Hispanic White pregnant people. High rates of police violence against White people may reflect the cumulative magnitude of punitive ideologies and practices in an area (rates of arrest, incarceration, surveillance, and non-fatal police violence), the effects of which are well documented in vulnerable Black, indigenous, and other communities of color and align with our findings among Black pregnant people. However, the potentially negative impacts of these practices on non-Hispanic White and high-income persons have largely been overlooked and may remain beyond those groups’ awareness. Moreover, it is possible given the history of racialized violence in the USA that state violence against persons of color comes to be expected and routine, such that state violence against White individuals stands out more in the minds of other White people to a greater effect.

A structural-psychological model of police violence developed by Devylder posits that police violence conceptually differs from other types of violence because of a unique combination of factors, including the proliferation of firearms, lack of accountability for police officers, the American legacy of racism, and structural stigma [30, 31]. However, framing these factors in the realm of public health requires the consideration of police-related violence as a reportable health condition which can be systematically surveilled, and thus acted upon with public health policies and interventions [4]. By appending police-related violence to existing public health monitoring mechanisms, improvements can be made in the surveillance and critical analysis of this public health issue, which are necessary to inform and develop efficacious interventions that address racial inequities in police violence and reproductive health [32]. Efforts must also be made to ensure that public safety and health are a priority in policing practices while also holding police agencies accountable for any unjust practices.

The strengths of this study lie in the analytic methods, including the use of state and year fixed effects adjusted models that control for differences in measured and unmeasured factors at multiple levels and temporal trends over the study timeframe. While another study has examined the effect of police violence on preterm birth [13], this paper is unique in its broad scope. We utilized national, population-level data, expanding on prior work in single jurisdictions. Our results also reiterate the importance of a health equity perspective through identification of racial differences in the exposure (police violence), the outcome (preterm birth), and stratification [33] since results varied by the race of the police-related fatality victims, as well as by maternal race. Additionally, by building upon a previous definition of police-related fatality with the inclusion of vehicle-related deaths, our findings underscore the importance of examining the effects of vehicle-related fatal police violence and a need for prevention efforts that include reducing thresholds for initiating traffic stops and car chases [24].

There are, however, some limitations to this study. While we explored the relationship between area-level police-related fatality rates at two geographic levels (MSA and counties within MSAs), it may be that the association between police violence and reproductive health is of greater relevance at a more spatially proximate level (neighborhood, for example). It may also be that spatial boundaries do little to prevent the spillover of psychosocial stress caused by police violence in cases that are increasingly covered on social media and local, state, and national news media outlets. We also lack data on non-fatal injury and other morbidities that result from interactions with police, which may occur more often and may also increase the risk of preterm birth. Further, because we linked annual exposure rates to birth records by year, we assume that all births in the year were exposed to the same rate of fatal violence, which may not be the case for births occurring earlier versus later in the year if rates changed over time. There is always the possibility of residual confounding, including from other dimensions of structural racism. Our findings on preterm birth also exclude impacts of fatal police violence that may have led to pregnancy loss [14], thus underestimating the full effects of fatal police violence on pregnant people. Lastly, we are unable to identify the relevant period of exposure to police violence because we do not have information on maternal residence preceding the residential information provided on the birth record. Our findings could therefore be reflective of a life-course accumulation of exposure to police violence and weathering not observed in our dataset wherein the perinatal period is the subject [34].

## Conclusion

There is increasing awareness that the adverse consequences of fatal police violence extend beyond the victim or victims directly involved. A public health approach identifies fatal police violence as a social determinant of population health outcomes and inequities, including preterm birth. Continued attention to encounters between police and the public could inform future interventions for this growing societal concern.

### Supplementary Information

Below is the link to the electronic supplementary material.

Supplementary file1 (DOCX 18 KB)
