# Supplementary material for: Investigating the Black Birth Experience: A Race-Stratified Analysis of Preterm Birth Risk and Exposure to Metropolitan Statistical Area-Level Police-Related Deaths, US 2018–2019
Source: J Urban Health. 2024 May 16;101(3):464–72. doi: 10.1007/s11524-024-00871-x (PMC11190101; doi:10.1007/s11524-024-00871-x)
Supplement: Supplementary file 1 — Supplementary file1 (DOCX 18 KB) [file 11524_2024_871_MOESM1_ESM.docx]

| Supplemental Table 1: List of US Census Designated Metropolitan Statistical Areas Included in the Final Analytic Dataset (where Total Number of Police Related Deaths >0)” |
| --- |
| Metropolitan Statistical Area Name |
| Abilene, TX |
| Akron, OH |
| Albany, GA |
| Albany-Lebanon, OR |
| Albany-Schenectady-Troy, NY |
| Albuquerque, NM |
| Alexandria, LA |
| Allentown-Bethlehem-Easton, PA-NJ |
| Altoona, PA |
| Amarillo, TX |
| Anchorage, AK |
| Anniston-Oxford, AL |
| Appleton, WI |
| Asheville, NC |
| Athens-Clarke County, GA |
| Atlanta-Sandy Springs-Alpharetta, GA |
| Atlantic City-Hammonton, NJ |
| Auburn-Opelika, AL |
| Augusta-Richmond County, GA-SC |
| Austin-Round Rock-Georgetown, TX |
| Bakersfield, CA |
| Baltimore-Columbia-Towson, MD |
| Bangor, ME |
| Baton Rouge, LA |
| Battle Creek, MI |
| Beaumont-Port Arthur, TX |
| Beckley, WV |
| Bellingham, WA |
| Bend, OR |
| Billings, MT |
| Binghamton, NY |
| Birmingham-Hoover, AL |
| Blacksburg-Christiansburg, VA |
| Bloomington, IN |
| Boise City, ID |
| Boston-Cambridge-Newton, MA-NH |
| Boulder, CO |
| Bowling Green, KY |
| Bremerton-Silverdale-Port Orchard, WA |
| Bridgeport-Stamford-Norwalk, CT |
| Brownsville-Harlingen, TX |
| Brunswick, GA |
| Buffalo-Cheektowaga, NY |
| Burlington, NC |
| Burlington-South Burlington, VT |
| California-Lexington Park, MD |
| Canton-Massillon, OH |
| Cape Coral-Fort Myers, FL |
| Carson City, NV |
| Casper, WY |
| Champaign-Urbana, IL |
| Charleston, WV |
| Charleston-North Charleston, SC |
| Charlotte-Concord-Gastonia, NC-SC |
| Charlottesville, VA |
| Chattanooga, TN-GA |
| Cheyenne, WY |
| Chicago-Naperville-Elgin, IL-IN-WI |
| Chico, CA |
| Cincinnati, OH-KY-IN |
| Clarksville, TN-KY |
| Cleveland, TN |
| Cleveland-Elyria, OH |
| Coeur d'Alene, ID |
| Colorado Springs, CO |
| Columbia, SC |
| Columbus, GA-AL |
| Columbus, OH |
| Corpus Christi, TX |
| Crestview-Fort Walton Beach-Destin, FL |
| Cumberland, MD-WV |
| Dallas-Fort Worth-Arlington, TX |
| Dalton, GA |
| Danville, IL |
| Daphne-Fairhope-Foley, AL |
| Davenport-Moline-Rock Island, IA-IL |
| Dayton-Kettering, OH |
| Deltona-Daytona Beach-Ormond Beach, FL |
| Denver-Aurora-Lakewood, CO |
| Des Moines-West Des Moines, IA |
| Detroit-Warren-Dearborn, MI |
| Dover, DE |
| Duluth, MN-WI |
| Durham-Chapel Hill, NC |
| East Stroudsburg, PA |
| Eau Claire, WI |
| El Centro, CA |
| El Paso, TX |
| Elizabethtown-Fort Knox, KY |
| Elmira, NY |
| Enid, OK |
| Erie, PA |
| Eugene-Springfield, OR |
| Evansville, IN-KY |
| Fairbanks, AK |
| Fargo, ND-MN |
| Farmington, NM |
| Fayetteville, NC |
| Fayetteville-Springdale-Rogers, AR |
| Flagstaff, AZ |
| Flint, MI |
| Florence, SC |
| Florence-Muscle Shoals, AL |
| Fort Collins, CO |
| Fort Smith, AR-OK |
| Fort Wayne, IN |
| Fresno, CA |
| Gadsden, AL |
| Gainesville, FL |
| Gainesville, GA |
| Gettysburg, PA |
| Grand Forks, ND-MN |
| Grand Island, NE |
| Grand Junction, CO |
| Grand Rapids-Kentwood, MI |
| Grants Pass, OR |
| Great Falls, MT |
| Greeley, CO |
| Green Bay, WI |
| Greensboro-High Point, NC |
| Greenville, NC |
| Greenville-Anderson, SC |
| Gulfport-Biloxi, MS |
| Hagerstown-Martinsburg, MD-WV |
| Hammond, LA |
| Hanford-Corcoran, CA |
| Harrisburg-Carlisle, PA |
| Harrisonburg, VA |
| Hartford-East Hartford-Middletown, CT |
| Hattiesburg, MS |
| Hickory-Lenoir-Morganton, NC |
| Hilton Head Island-Bluffton, SC |
| Hinesville, GA |
| Homosassa Springs, FL |
| Houma-Thibodaux, LA |
| Houston-The Woodlands-Sugar Land, TX |
| Huntington-Ashland, WV-KY-OH |
| Huntsville, AL |
| Idaho Falls, ID |
| Indianapolis-Carmel-Anderson, IN |
| Ithaca, NY |
| Jackson, MI |
| Jackson, MS |
| Jackson, TN |
| Jacksonville, FL |
| Jacksonville, NC |
| Janesville-Beloit, WI |
| Jefferson City, MO |
| Johnson City, TN |
| Jonesboro, AR |
| Joplin, MO |
| Kahului-Wailuku-Lahaina, HI |
| Kalamazoo-Portage, MI |
| Kansas City, MO-KS |
| Kennewick-Richland, WA |
| Killeen-Temple, TX |
| Kingsport-Bristol, TN-VA |
| Kingston, NY |
| Knoxville, TN |
| Kokomo, IN |
| Lafayette, LA |
| Lafayette-West Lafayette, IN |
| Lake Charles, LA |
| Lake Havasu City-Kingman, AZ |
| Lakeland-Winter Haven, FL |
| Lancaster, PA |
| Lansing-East Lansing, MI |
| Laredo, TX |
| Las Cruces, NM |
| Las Vegas-Henderson-Paradise, NV |
| Lawton, OK |
| Lebanon, PA |
| Lewiston, ID-WA |
| Lewiston-Auburn, ME |
| Lexington-Fayette, KY |
| Lincoln, NE |
| Little Rock-North Little Rock-Conway, AR |
| Logan, UT-ID |
| Longview, TX |
| Longview, WA |
| Los Angeles-Long Beach-Anaheim, CA |
| Louisville/Jefferson County, KY-IN |
| Lubbock, TX |
| Lynchburg, VA |
| Macon-Bibb County, GA |
| Madera, CA |
| Madison, WI |
| Manchester-Nashua, NH |
| Manhattan, KS |
| Mansfield, OH |
| McAllen-Edinburg-Mission, TX |
| Medford, OR |
| Memphis, TN-MS-AR |
| Merced, CA |
| Miami-Fort Lauderdale-Pompano Beach, FL |
| Midland, MI |
| Midland, TX |
| Milwaukee-Waukesha, WI |
| Minneapolis-St. Paul-Bloomington, MN-WI |
| Missoula, MT |
| Mobile, AL |
| Modesto, CA |
| Monroe, LA |
| Montgomery, AL |
| Morgantown, WV |
| Morristown, TN |
| Mount Vernon-Anacortes, WA |
| Myrtle Beach-Conway-North Myrtle Beach, SC-NC |
| Napa, CA |
| Nashville-Davidson--Murfreesboro--Franklin, TN |
| New Bern, NC |
| New Orleans-Metairie, LA |
| New York-Newark-Jersey City, NY-NJ-PA |
| North Port-Sarasota-Bradenton, FL |
| Norwich-New London, CT |
| Ocala, FL |
| Odessa, TX |
| Ogden-Clearfield, UT |
| Oklahoma City, OK |
| Olympia-Lacey-Tumwater, WA |
| Omaha-Council Bluffs, NE-IA |
| Orlando-Kissimmee-Sanford, FL |
| Oshkosh-Neenah, WI |
| Owensboro, KY |
| Oxnard-Thousand Oaks-Ventura, CA |
| Palm Bay-Melbourne-Titusville, FL |
| Panama City, FL |
| Pensacola-Ferry Pass-Brent, FL |
| Peoria, IL |
| Philadelphia-Camden-Wilmington, PA-NJ-DE-MD |
| Phoenix-Mesa-Chandler, AZ |
| Pine Bluff, AR |
| Pittsburgh, PA |
| Pocatello, ID |
| Port St. Lucie, FL |
| Portland-South Portland, ME |
| Portland-Vancouver-Hillsboro, OR-WA |
| Poughkeepsie-Newburgh-Middletown, NY |
| Prescott Valley-Prescott, AZ |
| Providence-Warwick, RI-MA |
| Provo-Orem, UT |
| Pueblo, CO |
| Punta Gorda, FL |
| Racine, WI |
| Raleigh-Cary, NC |
| Rapid City, SD |
| Redding, CA |
| Reno, NV |
| Richmond, VA |
| Riverside-San Bernardino-Ontario, CA |
| Roanoke, VA |
| Rochester, MN |
| Rochester, NY |
| Rockford, IL |
| Rocky Mount, NC |
| Rome, GA |
| Sacramento-Roseville-Folsom, CA |
| Saginaw, MI |
| Salem, OR |
| Salinas, CA |
| Salisbury, MD-DE |
| Salt Lake City, UT |
| San Antonio-New Braunfels, TX |
| San Diego-Chula Vista-Carlsbad, CA |
| San Francisco-Oakland-Berkeley, CA |
| San Jose-Sunnyvale-Santa Clara, CA |
| Santa Fe, NM |
| Santa Maria-Santa Barbara, CA |
| Santa Rosa-Petaluma, CA |
| Savannah, GA |
| Scranton--Wilkes-Barre, PA |
| Seattle-Tacoma-Bellevue, WA |
| Sebastian-Vero Beach, FL |
| Sebring-Avon Park, FL |
| Shreveport-Bossier City, LA |
| Sioux City, IA-NE-SD |
| Sioux Falls, SD |
| South Bend-Mishawaka, IN-MI |
| Spartanburg, SC |
| Spokane-Spokane Valley, WA |
| Springfield, MA |
| Springfield, MO |
| Springfield, OH |
| St. Joseph, MO-KS |
| St. Louis, MO-IL |
| State College, PA |
| Stockton, CA |
| Syracuse, NY |
| Tallahassee, FL |
| Tampa-St. Petersburg-Clearwater, FL |
| Terre Haute, IN |
| Texarkana, TX-AR |
| The Villages, FL |
| Toledo, OH |
| Topeka, KS |
| Trenton-Princeton, NJ |
| Tucson, AZ |
| Tulsa, OK |
| Tuscaloosa, AL |
| Twin Falls, ID |
| Tyler, TX |
| Urban Honolulu, HI |
| Utica-Rome, NY |
| Valdosta, GA |
| Vallejo, CA |
| Vineland-Bridgeton, NJ |
| Virginia Beach-Norfolk-Newport News, VA-NC |
| Visalia, CA |
| Waco, TX |
| Walla Walla, WA |
| Washington-Arlington-Alexandria, DC-VA-MD-WV |
| Waterloo-Cedar Falls, IA |
| Wausau-Weston, WI |
| Wheeling, WV-OH |
| Wichita, KS |
| Williamsport, PA |
| Wilmington, NC |
| Winston-Salem, NC |
| Worcester, MA-CT |
| Yakima, WA |
| York-Hanover, PA |
| Youngstown-Warren-Boardman, OH-PA |
| Yuba City, CA |
| Yuma, AZ |
